# Black carbon derived PET plastic bottle waste and rice straw for sorption of Acid Red 27 dye: Machine learning approaches, kinetics, isotherm and thermodynamic studies

**DOI:** 10.1371/journal.pone.0290471

**Published:** 2023-08-23

**Authors:** Tapos Kumar Chakraborty, Lamia Tammim, Khandakar Rashedul Islam, Md. Simoon Nice, Baytune Nahar Netema, Md. Sozibur Rahman, Sujoy Sen, Samina Zaman, Gopal Chandra Ghosh, Asadullah Munna, Ahsan Habib, Khadiza Tul-Coubra, Himel Bosu, Monishanker Halder, Md. Aliur Rahman

**Affiliations:** 1 Department of Environmental Science and Technology, Jashore University of Science and Technology, Jashore, Bangladesh; 2 Department of Computer Science and Engineering, Jashore University of Science and Technology, Jashore, Bangladesh; 3 Department of Petroleum and Mining Engineering, Jashore University of Science and Technology, Jashore, Bangladesh; Universiti Teknologi Petronas: Universiti Teknologi PETRONAS, MALAYSIA

## Abstract

This study focuses on the probable use of PET waste black carbon (PETWBC) and rice straw black carbon (RSBC) as an adsorbent for Acid Red 27 (AR 27) adsorption. The prepared adsorbent is characterized by FE-SEM and FT-IR. Batch adsorption experiments were conducted with the influencing of different operational conditions namely time of contact (1–180 min), AR 27 concentration (5–70 mg/L), adsorbent dose (0.5–20 g/L), pH (2–10), and temperature (25–60°C). High coefficient value [PETWBC (*R*^*2*^ = 0.94), and RSBC (R^2^ = 0.97)] of process optimization model suggesting that this model was significant, where pH and adsorbent dose expressively stimulus removal efficiency including 99.88, and 99.89% for PETWBC, and RSBC at pH (2). Furthermore, the machine learning approaches (ANN and BB-RSM) revealed a good association between the tested and projected value. Pseudo-second-order was the well-suited kinetics, where Freundlich isotherm could explain better equilibrium adsorption data. Thermodynamic study shows AR 27 adsorption is favourable, endothermic, and spontaneous. Environmental friendliness properties are confirmed by desorption studies and satisfactory results also attain from real wastewater experiments. Finally, this study indicates that PETWBC and RSBC could be potential candidates for the adsorption of AR 27 from wastewater.

## Introduction

Organic dyes are extensively used as colouring agents in many industries including textiles, leather, paint, cosmetics, rubber, paper, ceramics, varnishes, pulp mills, ink, plastics, pharmaceuticals, and tanneries [1]. Globally, 10,000 textile dyes are available while over 7×105 tones are manufactured yearly and 75% of it is used in the textile industry [2]. It is estimated that more than 50% of the used dyes do not fix the fabrics, and these are released as coloured effluent from industrial manufacturing and operation steps [2, 3]. Quick industrial development is highly responsible for environmental degradation by releasing huge toxic effluents and now a day it is considered a global issue, especially for developing countries [4–6]. As dyes are chemically and thermally stable so they must be treated properly before discharging into an aquatic environment. Colourful wastewater changes the ecosystem equilibrium by decreasing photosynthesis activities, enhancing colour bleaching, impeding light penetration, inhibiting fauna growth rate, producing micro toxins, and increasing metal chelating [7, 8]. Additionally, exposing elevated levels of dyes and their associated byproduct can create several health hazards to humans such as skin irritation, eye burns, and damage to the kidney, liver, and central nervous system [9, 10] and is also considered mutagenic and carcinogenic. As dyes are chemically and thermally stable so it must be treated properly before discharging into an aquatic environment. Based on solubility, dyes are categorized into three types: anionic, cationic, and nonionic. Among them, azo dyes are a versatile class of organic dyes and include 60–70% of all dye production [1, 8]. Acid red 27 (AR 27), is a modified red-azo dye compound extensively utilize as a food dye and to colour cosmetics in many industries due to ease of application, colour brightness, and excellent binding capacity, but its elimination is challenging for varied features (e.g., aromatic ring structure, resistance to degradation, azo link and highly stable to heat and light) [11]. Therefore, wastewater must be free from toxicants before releasing environments for marinating environmental quality and developing an eco-friendly industry. There are numerous treatment approaches exist for the elimination of dyes from polluted water, such as UV and ozonation, coagulation and flocculation, chemical oxidation processes, membrane separation, reverse osmosis, ion exchange, and photolysis [12–17]. Furthermore, several treatment methods are either costly or have several drawbacks [18]. Barakat [19] has extensively explored the benefits and limitations of various treatment methods. Adsorption is mostly using effluent treatment techniques for ease of operation and design, cost-effectiveness, greater performance, source availability, lower sludge, flexibility, and insensitivity to hazardous elements [1, 8]. The usability of marketable activated carbon is reducing due to high cost [18]. In this favour, many wastes and biomaterials were used as adsorbents for both inorganic and organic pollutants, broadly reviewed by Bhattacharjee et al. [20]. Nowadays numerous researchers are trying to develop novel, low-cost, and ecologically viable adsorbents with high performance from waste products for the elimination of hazardous substances from wastewater. Consequently, more recent studies have investigated the use of numerous waste biomasses, such as agricultural wastes [21] and sludge [22], to prepare low/no-cost activated carbon. Biochar (thermal conversion of carbon-containing biomass product) is a favourable substitute for commercial activated carbon for cost effectiveness, high adsorption area, and good adsorption capability [23]. Literature review indicates that most of the study uses biomaterials for dye adsorption [24], Gracilaria corticate algae [25], Azolla filiculoides aquatic fern [26], and advanced materials including single-walled carbon nanotubes [27], using titanium dioxide nanoparticles/graphene oxide nanocomposite [28, 29], chitosan-glyoxal/TiO2 nanocomposite [30], surfactant-modified zeolite [31], surfactant-modified bentonite [32], therefore, this present study tried to develop black carbon from plastic discard materials (eg. PET bottles) and rice straws using a simple and low-cost process. Bangladesh is an agricultural country where rice is widely cultivated; there is a high potential to produce biochar from rice straw for the management of waste material. Additionally, Polyethylene terephthalate (PET) is a widely used polymer around the world for its particular properties such as being lightweight, cost-effective, clearness, a good insulator, exceptional flexibility, and easy to handle [33, 34], so yearly huge amount of plastic waste is generating that is highly concern able for the environment [35]. PET is the widely produces public and industrial discarded materials which have no potential beneficiary application. Therefore, PET waste management is considered a global burden, especially in the developing country [36], where burning and landfilling are the commonly used disposal methods, which are highly responsible for environmental pollution by emitting toxic gaseous pollutants and polluting landfilling surrounding ecosystem (eg. aquatic and terrestrial) [37]. Though recycling is another method for PET waste management instead of burning and landfilling, this method is not effective due to a lack of technical difficulties and lower economic return, in this viewpoint activated carbon preparation from PET waste would be a substitute technique as compared to other techniques [33]. Providentially, PET discard products contain high carbon and lower impurities, which act as a new window for developing activated carbon and pollutants adsorbing agents [38]. In the experimental study, process modelling and optimization are very important to improve the system performance but conventional methods could optimize a single parameter at a time which increases experimental time and cost [39]. Recently, many researchers are paid attention to applying artificial neural networks (ANN) and Box- Behnken design Response surface methodology (BBD-RSM) for optimizing and modelling the diverse experimental parameters at a time which enhances the system performance [40]. Those machine learning approaches are a reliable and powerful tool which helps to overwhelm the system limitations and to assess actual results using experimental data. It is a soft computing technique where required results could be achieved via alternating network weights [39]. BBD-RSM is mostly using the prevailing statistical tool for process optimization [41, 42] and it reduces operational cost, manpower, and process time [30, 43, 44]. So, it does not need any particular understanding of the physical/chemical procedure that moves the system. Nowadays, ANN-RSM-based approaches have been used for the diverse area of environmental engineering [45–47] but very few studies have been conducted for textile dye adsorption. The ultimate objectives of this study were (i) to investigate the removal performance of black carbon for toxic dye (eg. AR 27) from wastewater using diverse operational conditions such as pH, time of contact, diverse dye concentration, temperature, and adsorbent dose via ANN-RSM modelling; (ii) to explore the adsorption mechanism using diverse models namely, isotherm, kinetic, and thermodynamic; (iii) to assess the practical application and further contamination tendency of adsorbent using real wastewater treatment and desorption experiments, respectively.

## Materials and methods

### Materials and reagents

In the entire experiment, using every chemical and reagent was laboratory-grade, purchased from Sigma-Aldrich (Germany), namely Acid Red 27 (85-95%). Lab-grade de-ionised water was used for the whole study.

### Adsorbent preparation and characterization

Portable drinking water bottles were selected as PET plastic waste, and rice straw was collected from the local area, Jashore, Bangladesh. Firstly, the waste bottle and rice straw were washed with deionized water to remove visible impurities, cut into small sizes, and dried at 80°C in an electric oven (Oven DSO-500D, Taiwan) until getting moisture free, and cool at ambient conditions. After that, PETWBC and RSBC were produced by burning in an electric furnace at 600°C for 100 min and 500°C for 15 min retention time, respectively. Then carbonize products were crushed and preferred size portions (0.5 to 1.0 mm) were collected through a conventional sieve. Finally, store it in a sealed glass bottle for next experimental uses. Synthesize graphene was characterized using FT-IR, and SEM. The surface morphology of prepared graphene was investigated with FE-SEM, Zeiss Sigma 300, Carl Zeiss, Germany, at 10 kV voltages. Before analysis, the graphene powder was coated with gold for better imaging and to escape the addition of native electrical charges. The surface chemistry was investigated by FTIR (Nicolet™ iS20, Thermo Scientific, USA), where the recorded spectra range varied from 400–4,000 cm^-1^ with 50 scans attained at 4 cm^-1^ resolution. Demirhan [4] provided a method that was applied for point of zero charges (pHpzc) assessment.

### Adsorption experiments

The required quantity of dye powder is dissolved in distilled water for preparing 1000 ppm stock solution and kept the stock solutions pH was less than 2.0 using HNO_3_, then successive dilution approaches were used to prepare the preferred working solution from the stock solution. Adsorption of AR 27 onto PETWBC and RSBC was carried out in a batch mode at 200 rpm using the following operational conditions: pH (2–10), time of contact (1–180 min), temperature (25–60 _0_C), graphene dose (0.5–20 g/L), and AR 27 concentration (5–70 mg/L). For pH adjustment, 0.1 N acid (HNO_3_) and base (NaOH) solution was used. After a certain time, samples were taken, and, filtered for AR 27 concentration analysis using a UV-visible spectrophotometer (HACH DR 3900, USA) at 520 nm wavelength. A duplicate test was conducted to gating accurate results. The total AR 27 adsorption rate and removal efficiency was estimated using Eqs 1 and 2, respectively. All laboratory experiments were conducted in the Environmental Chemistry Lab, Department of Environmental Science and Technology, Jashore University of Science and Technology, Jashore 7408, Bangladesh after taking permission and following all procedures.


$$q_{e}=\frac{{(C}_{0}-C_{e})V}{m_{s}}$$ (1)

$$R(\%)=\frac{\left(C_{0}-C_{e}\right)}{C_{0}}\times 100$$ (2)

Where,

C_o_ = initial AR 27 concentration (mg/L)

C_e_ = equilibrium AR 27 concentrations (mg/L),

q_e_ = amount of AR 27 adsorbed (mg/g)

V = volume of liquid solution (L)

m_s_ = graphene mass (g).

### Adsorption isotherm and kinetic experiments

Adsorption isotherm studies were carried out into 250 mL dye solutions of varied dye concentrations (5 to 70 mg/L), at pH 2, where 10 g/L adsorbent dose (PETWBC and RSBC) was added and stirred the solution at 200 rpm with ambient temperature for 150 min. While kinetics experiments were run in 300 mL dye solution at a fixed concentration (20 mg/L) and kept the other condition constant, then samples were taken out after following time intervals 1, 5, 7, 10, 15, 20, 30, 60, 90, 120, 150, and 180 min, filtered, and analyzed. This study applied Langmuir and Freundlich for equilibrium data modelling while pseudo-first-order and pseudo-second-order were used for kinetic modelling, detailed presented in S1 Table in S1 File.

### Error analysis

Error analysis is applied for every used model to assess the level of error as compared with obtained and experimental results. In this study, residual sum square (RSS) chi-square (χ^2^) tests, and root means square errors (RMSE) error analysis test (Eq 4–6) were calculated to determine which adsorption isotherm and kinetic models fitted to experimental data [1]. A smaller error value denotes the model that fits the data the best. Eqs 3–5 describe the formula for determining the best-fit model.

$$RSS=\sum ({q_{exp}-q_{cal})}^{2}$$ (3)

$$\chi 2=\sum \frac{({q_{exp}-q_{cal})}^{2}}{q_{cal}}$$ (4)

$$RMSE=\sqrt{\frac{\sum_{i=1}^{n}({q_{exp}-q_{cal})}^{2}}{n}}$$ (5)

Where q_exp_ is the observed experimental adsorption data (mg/g) from the kinetic models, q_cal_ is the calculated adsorption data (mg/g) from models, and n represents the number of data sets.

### Desorption study

For the desorption study, AR 27-loaded adsorbent was attained from adsorption isotherm experiments, filtered, and dried. Finally, the experiment was conducted in distilled water with diverse pH and stirring the solution at 200 rpm for 150 min. Eq 6 was applied for calculating the outcome.


$$Desorption\;(\%)=\frac{Mass\;of\;AR\;27\;desorbed\;(mg/L)}{Mass\;of\;AR\;27\;adsorbed\;(mg/L)}\times 100$$ (6)

### Adsorption thermodynamics

Thermodynamics is a vital parameter for adsorption study, where temperature variation is needed for conducting this study. In this study, Gibbs free energy change (ΔG), enthalpy (ΔH) and entropy (ΔS) are calculated by applying the following Eqs 7–9.

$$K_{d}=\frac{q_{e}}{c_{e}}$$ (7)

$$\Delta G=-RT\;\ln\;K_{d}$$ (8)

$${lnK}_{d}=\frac{\Delta S}{R}-\frac{\Delta H}{RT}$$ (9)

Where, T = temperature (K), K_d_ = equilibrium constant, R = universal gas constant (J mol^−1^ K^−1^)

### Process optimization

Box- Behnken designing (BBD) approach is an appropriate statistical tool widely used for process optimization, where the least number of experiments were applied to explore the probable association between the experimental parameters and their influences on the adsorbate adsorption [39]. This study uses three-level three factorial BBD where three factors are defined as X_1_ = pH, X_2_ = AR 27 concentration, and X_3_ = graphene dose and three levels are stated as upper (1), central (0), and lower (-1), detailed presented in Table 1. This model runs 17 experiments using Stat-Ease software (Design-Expert 13.0 trial version, Stat-Ease, Inc.). The following polynomial equation Eq 10 is used for BBD modelling.


$$Z=\beta_{0}+\sum_{I=1}^{n}\beta_{i}A_{i}+\sum_{i=n}^{n}\beta_{ij}A_{i}A_{j}+\sum_{i=1}^{n}\beta_{ii}A_{i}^{2}$$ (10)

Where Z is the projected response (dye adsorption (%); q_e_ (Cd), q_e_ (Pb), q_e_ (Cu), q_e_ (Zn), β_0_ = Constant, β_i_ = linear coefficient. β_ij_ = interface coefficients, β_ii_ = quadratic coefficients, and A_i_ and A_j_ = process variables.

**Table 1:** Independent variables, their experimental range and three levels of these variables for AR 27 dye adsorption using PETWBC, and RSBC.

Factors	Levels		
	-1	0	+1
X_1_: Solution pH	2	6	10
X_2_: AR 27 concentration (mg/L)	5	30	70
X_3_: Activated carbon ratio (g/L)	0.5	10	20

## Results and discussion

### Characterization of adsorbent

**Fig 1:**
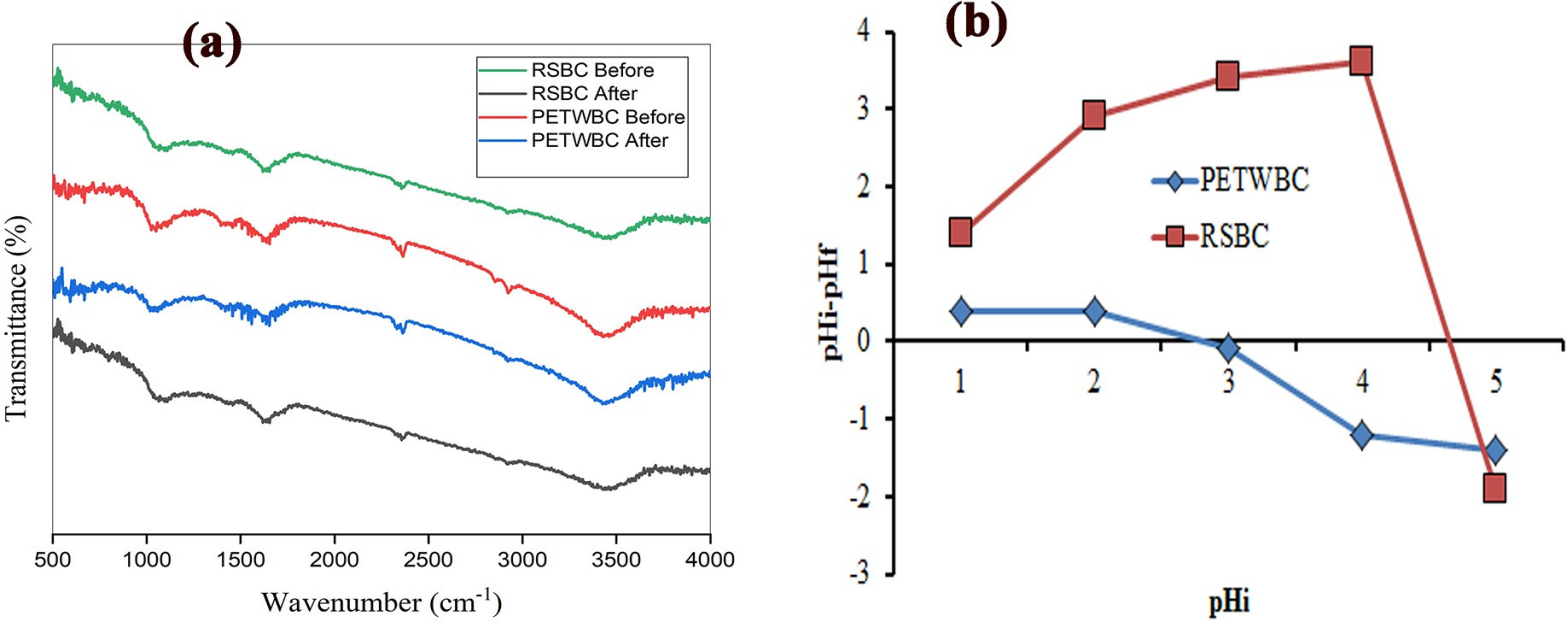
(a) FT-IR spectra of adsorbent (PETWBC, and RSBC), and (b) pH of point zero charge.

The pHpzc is a crucial component that delivers a clear concept regarding adsorbent surface charging and its relations with the target component. Fig 1B shows the curve of (pHi—pH_f_) vs pH_i_, where 2.99 and 4.7 were discovered as the pHpzc value for PETWBC, and RSBC respectively. According to the pHpzc concept, the graphene shows a positive attitude at solution pH< pHpzc value, conversely a negative attitude expresses at solution pH > pHpzc value. FTIR analysis represents the surface chemistry of graphene (before and after adsorption), where diverse functional groups influence the linkage between adsorbate and adsorbent, obtained results presented in S1a Fig in S1 File.

The adsorption peak nearby 1600 cm^−1^ matching the in-plane C = C vibration indicates the graphite properties, that is inborn properties of sp2 graphitic ingredients [48]. Raw adsorbent shows the following peaks at 3444, 2923, 2394, 1734, 1684, 1558, and 1059cm^−1^ (S1a Fig in S1 File), indicating the stretching vibration of OH [49], C-H [49], O = C = O [47], C = O [37], C = O, C-N, and C = O [50], respectively for PETWBC, while 3469, 2394, 1653, 1109, and 844 cm^−1^ shows OH [49], O = C = O [47], C = C [8, 51], C = O [52], C-Cl, respectively for RSBC [35]. Slight alterations were observed after AR 27 adsorption (S1a Fig in S1 File), while above mentioned adsorption peaks can link with the AR 27 molecules while more specifically hydroxyl group (OH) and heteroatoms highly regulate the linkage between adsorbent and adsorbate. A similar result was reported by El Essawy et al. [35] for acid and basic adsorption using graphene prepared from PET plastic bottle. The surface feature of both adsorbents (before and after adsorption) was estimated through SEM analysis, attained results presented in S1 Fig in S1 File. Raw adsorbent exhibits lots of pores and a rough surface which assists in holding adsorbate ions (S1a, S1c Fig in S1 File). PET waste-activated carbon contains lots of micropores and mesopores that are confirmed by Djahed et al. [33], Kumari et al. [53], and Sackey et al. [54]. While both adsorbent surfaces were fully covered with AR 27 dye molecules due to after adsorption (S1b, S1d Fig in S1 File).

### Adsorption behaviour

#### Effect of contact time

**Fig 2:**
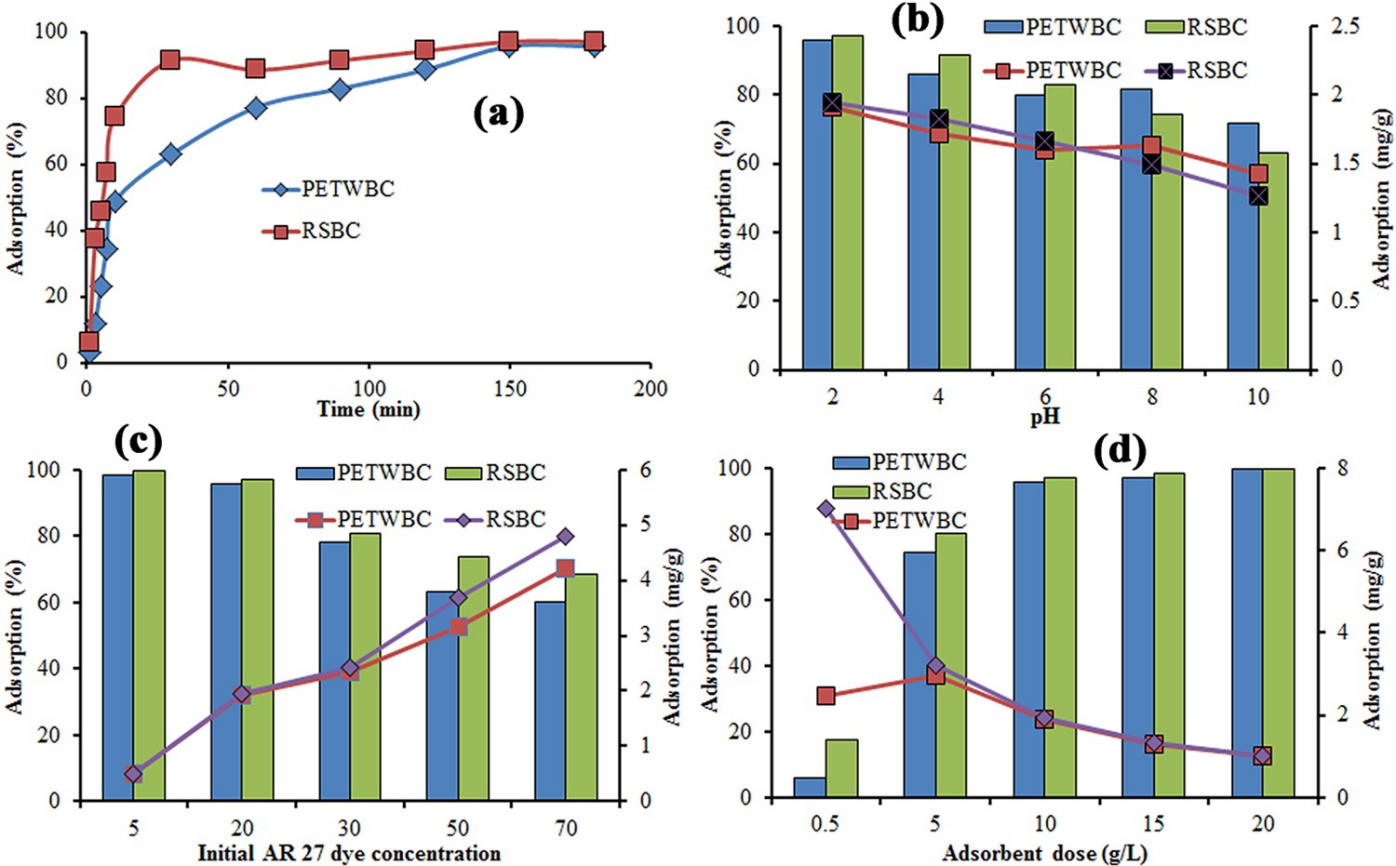
Adsorption of AR 27 dye using graphene at diverse operational factors (a) contact time (b) pH; (c) initial dye concentration; (d) Adsorbent dose [adsorbent dose = 10 g/L; dye concentration = 20 mg/L; optimum pH = 2), Temperature = 25°C; equilibrium contact time = 150 min].

To assess the adsorption behaviour and equilibrium adsorption capabilities of PETWBC and RSBC for AR 27 adsorption was examined at diverse contact times, presented in Fig 2. These experiments were evaluated using an AR27 concentration of 20 mg/L, temperature of 25°C, pH equal to 2 and mass of PETWBC and RSBC (10 g/L). Generally, three steps were involved during the adsorption process, as illustrated in (Fig 2A). Mainly three stages controlled the whole adsorption procedure: (1) quick adsorption was achieved at an early stage (1–10 min) due to bulk concentration of dye ions and huge vacant space on the adsorbent surface, (2) after 10–150 min the adsorption efficiency are turning into slow due to declining of existing binding sites with time movement, and (3) finally, the adsorption efficiency goes to comparatively very low within 150–180 min due to blocking of almost all vacant space (outer and inner site) on the adsorbent surface [55]. So, adsorption researched equilibrium in 150 minutes, which was chosen as the equilibrium contact time for further experiments. Sackey et al. [54] provide a similar explanation for the adsorption of basic red dyes using bamboo and rice straw biochar.

#### Effect of pH

For adsorption science, pH is the most significant factor which stimulates dye ionization rate, change of adsorbent inner and outer binding sites, and shifting of adsorbent surface charge [6]. Fig 2B shows the adsorption performance of AR 27 using PETWBC and RSBC at varied solution pH 2 to 10. The removal of AR 27 decreased (PETWBC = 95–71%; and RSBC = 97–62%) with rising solution pH (2–10), (Fig 2B), because the surfaces of adsorbents were deprotonating, so electrostatic repulsion between absorbate and adsorbent also increased at rising pH. The adsorbent surface becomes positive in a highly acidic environment due to the influence of oxygen-linked functional groups, where high proton clouds enrich electrostatic attraction between adsorbate and adsorbent, resulting in higher AR 27 removal. Therefore pH 2 was selected as the optimum pH for AR 27 adsorption study. Similar results for the adsorption of crystal violet dye onto modified rice husk were reported by Homagai et al. [16].

#### Effect of initial dye concentration

To investigate the performance of adsorbent for AR 27 adsorption with diverse concentrations (5–70 mg/L) an experiment was conducted where other factors are kept constant (t = 25°C, contact time = 150 min, pH = 2, dose = 10 g/L). Fig 2C shows that with rising AR 27 concentration (5 to 70 mg/L), the elimination performance of AR 27 (PETWBC = 98–60%; and RSBC = 99–68%) (Fig 2C) decrease due to saturating adsorbent external surface at a fixed dose, where, AR 27 dye molecules cover the vacant space. On the other hand, the adsorption capacity of the adsorbent increased (PETWBC = 0.50–4.20 mg/g, and RSBC = 0.49–4.78 mg/g) due to a higher interface between AR 27 dye molecules and adsorbent active sites with rising AR 27 dye concentration, where high diving force led to overcome the mass transfer between liquid and solid phases [56].

#### Effect of adsorbent dose

Pollutant elimination from wastewater is suggestively influenced by adsorbent dosage [8]. To assess the suitable adsorbent dose, batch adsorption experiments of AR 27 onto graphene were conducted at 25 ± 2°C, with different adsorbent doses (0.5–20 g/L) and keeping the other condition constant (pH = 2, AR 27 concentration = 20 mg/L, CT = 150 min). Fig 2D reveals that AR 27 removal percentage, improved with rising adsorbent doses (0.5–20 g/L) for AR 27 (PETWBC = 6–99%, and RSBC = 17–99%), respectively, due to lots of active exchangeable sites on the adsorbent surface, resulting in greater adsorption [1]. While the adsorption rate gradually reduces (PETWBC = 2.45–0.99 mg/g, and RSBC = 7.00–1.00 mg/g) with rising adsorption dose (0.5–20 g/L) might be the vying or coincide (e.g., assemblage) of AR 27 dye molecules onto the adsorbent. A related elucidation was given by Chakraborty et al. [6].

#### Kinetic models and adsorption mechanism

Adsorption kinetics is needed to design an effluent treatment plant unit, where adsorbate could be removed pollutants at a certain rate. In the present study, two commonly uses kinetics models such as Lagergren’s pseudo-first-order, and Ho’s pseudo-second-order applied for AR 27 adsorption onto PETWBC and RSBC. The model accuracy depends on the high correlation coefficient *(*R^2^) and lower error values (*RSS*, *chi-square (χ*^*2*^*) and RMSE) of the model*, and the applied model parameters are represented in Table 2. The pseudo-second-order kinetic model shows higher R^2^ and lower RSS, χ^2^, and RMSE values for AR 27 adsorption as compared with pseudo-first-order kinetic, additionally, the calculated (*q*_*e*, *cal*_) value from the pseudo-second-order kinetic model also corresponds to the experimental value (*q*_*e*,*exp*_), indicating that pseudo-second-order was best fitted kinetic model for adsorption data.

**Table 2:** Kinetic parameters for AR 27 adsorption onto PETWBC and RSBC.

Models	Parameters	RSBC	PETWBC
	q_e,exp_ (mg/g)	1.941	1.912
Pseudo-first order	q_e,cal_ (mg/g)	0.975	0.979
	K_1_ (min^–1^)	0.113	0.391
	R^2^	0.778	0.962
	RSS	5.36E+17	1.56E+61
	χ2	4.47E+16	1.3E+60
	RMSE	2.12E+08	1.14E+30
Pseudo-second order	q_e,cal_ (mg/g)	2.012	2.124
	K_2_ (g/mg/ min)	0.072	0.022
	H (mg/g/ min)	0.292	0.102
	R^2^	0.997	0.993
	RSS	0.406	0.119
	χ2	0.026	0.009
	RMSE	0.183	0.099
Intraparticle diffusion (1^st^ stage)	K_diff_ (mg/gmin^0.5^)	0.376	0.093
	C (mg/g)	0.352	0.417
	R^2^	0.961	0.981
Intraparticle diffusion (2^nd^ stage)	K_diff_ (mg/gmin^0.5^)	0.093	0.019
	C (mg/g)	0.742	1.671
	R^2^	0.975	0.706

**Fig 3:**
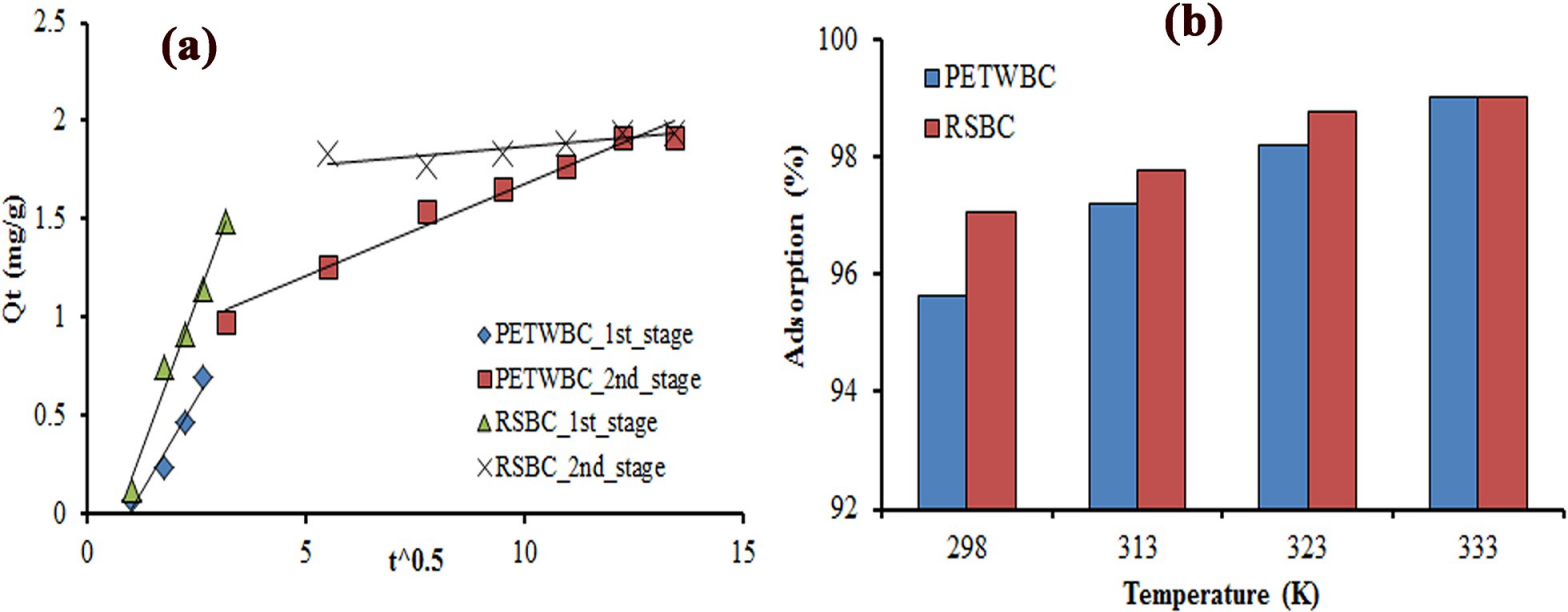
(a) Intraparticle diffusion plot, and (b) Effect of temperature for AR 27 dye adsorption using adsorbent (PETWBC, and RSBC).

The experimental data were also evaluated by intraparticle (IP) diffusion to determine the diffusion mechanisms. The two-stage of the IP plot did not pass through the origin (Fig 3), representing that exterior diffusion was the rate-limiting step as interior diffusion for AR 27 adsorption using adsorbent, which may happen at the same time. Salih et al. [57] observed related findings in their adsorption study.

#### Adsorption isotherm modelling

The linkage between adsorbate and adsorbent in an adsorption study is well explained by adsorption isotherms. In this present study, Langmuir and Freundlich’s isotherms were used, which are usually useful in the solid/liquid system, presented in Table 3. Freundlich was the best-fitted isotherm for AR 27 adsorption onto both adsorbents due to its higher correlation coefficient value (PETWBC: *R*^*2*^ = 0.964, RSBC: *R*^*2*^ = 0.959), lower error (PETWBC: RSS = 15.787, RSBC: RSS = 0.809), and chi-square value (PETWBC: *χ*^*2*^ = 3.157; RSBC: *χ*^*2*^ = 0.161) and (PETWBC: RMSE = 1.776, RSBC: RMSE = 0.402) as compare Langmuir isotherm (*R*^*2*^ = 0.947, 0.935; RSS = 20.353and 2.513; RSME = 2.015and 0.708; *x*^*2*^ = 4.070, and 0.502 for PETWBC and RSBC, respectively) (Table 3), showing multi-layer adsorption onto PETWBC and RSBC.

**Table 3:** Isotherm parameters for adsorption of AR 27 onto PETWBC and RSBC.

Isotherm models	Parameters	PETWBC	RSBC
Langmuir	q_max_ (mg/g)	4.108	4.840
	b (L/mg)	1.282	0.450
	R_L_	0.134–0.011	0.30–0.031
	R^2^	0.947	0.935
	RSS	20.353	2.513
	χ^2^	4.070	0.502
	RMSE	2.015	0.708
Freundlich	K_F_ (mg/g) (L/mg)^1/n^	2.851	2.017
	n	3.921	4.144
	R^2^	0.964	0.959
	RSS	15.787	0.809
	χ^2^	3.157	0.161
	RMSE	1.776	0.402
Temkin	A_T_ (L/mg)	93.838	72.685
	B_T_ (KJ/mol)	5.508	4.556
	R^2^	0.895	0.873
Dubinin-Radushkevich (D-R)	q_m_ (mg/g)	2.836	3.139
	β (mol^2^ K/J^2^))	1.97E-02	2.1E-02
	E (KJ/mol)	5.037	4.879
	R^2^	0.887	0.872

The monolayer maximum adsorption capacity of AR 27 was 4.108, and 4.840 mg/g for PETWBC and RSBC, respectively (Table 3). The *R*_*L*_ values of graphene were between 0 and 1, representing that AR 27 adsorption onto adsorbent was appropriate under the studied experimental conditions. Conversely, the value of adsorption intensity (*n*) was higher than 1 and higher *K*_*F*_, demonstrating that the adsorption process was promising for AR 27 adsorption from aqueous solutions using PETWBC AND RSBC (Table 3). Temkin and D-R isotherm models show that the adsorption is physical, where both the model parameter values were less than the guideline value (B_T_ < 8 KJ), and (E < 8 KJ/mol) (Table 3), respectively [31, 32]. This study outcome also correspondence with El Essawy et al. [35] and Zaman et al. [18] findings. Additionally, the performance of AR 27 adsorption using PETWBC and RSBC is comparable with other adsorbents, presented in Table 4.

**Table 4:** Adsorption performance of adsorbent (PETWBC, and RSBC) as compared with other adsorbents.

Adsorbents	Maximum adsorption capacity (mg/g)	Optimum dose (g/L)	Concentration range (mg/L)	pH	References
Swietenia mahagoni bark activated carbon	6.071	1–30	10–100	3	[12]
Hen feather	6.020	3–25	5–50	7	[56]
Carbonized microplastic particles	5.678	1–20	5–70	3	[58]
Mahagoni (Swietenia mahagoni) Bark Charcoal	5.402	1–20	5–70	3	[1]
Rice straw black carbon	4.840	0.5–20	5–70	2	This study
PET waste black carbon	4.108	0.5–20	5–70	2	This study
Banana peel	3.880	0.04–0.5	5–25	4	[59]
Mahagoni (Swietenia mahagoni) Wood charcoal	3.806	1–20	5–70	3	[1]
Coarse grinded wheat straw	3.820	-	-	-	[60]
Fine grinded wheat straw	2.230	-	-	4	[60]

#### Adsorption thermodynamics studies

The thermodynamic study represents the role of temperature for adsorption, the nature of the linkage between adsorbate and adsorbent, direction and mechanism of reaction with changing the experimental temperature [58]. These study results are presented in Table 5, and Fig 3B. A Van’t Hoff plot of lnkd vs 1/T made a straight line (not shown in the figure) with *R*^*2*^ values of 0.956, and 0.943 for PETWBC, and RSBC, respectively. Table 5 shows that negative values of ∆G^0^ demonstrate that AR 27 adsorption processes are thermodynamically spontaneous and useful [35].

**Table 5:** Thermodynamic modelling of adsorbent for AR 27 adsorption using PETWBC and RSBC.

Adsorbent	Temperature (K)	ΔG^0^ (KJ/mol)	ΔH^0^ (KJ/mol)	−ΔS^0^ (J/mol/K)	R^2^
PETWBC	298	-7.642	35.519	144.015	0.956
	313	-9.218			
	323	-10.712			
	333	-12.818			
RSBC	298	-8.651	28.088	122.631	0.943
	313	-9.821			
	323	-11.733			
	333	-12.818			

The endothermic type of adsorption is confirmed by the positive result of ∆H^0^. Lower ∆H^0^ value (< 40 kJ/mol) recommends that physisorption is the main mechanism for AR 27 adsorption [32], besides the adsorption rate increase with rising temperature, and no remarkable deviations were found after 298 K (25°C) (Fig 3B). Therefore, the next experiments were conducted at this temperature. The positive ∆S^0^ also suggests that physical alterations happen on the adsorbent during dye uptake through the ion exchange process. Sackey et al. [54] detect the endothermic reaction for basic red dye adsorption using bamboo and rice straw adsorbent.

#### Box–Behnken design and regression model

**Fig 4:**
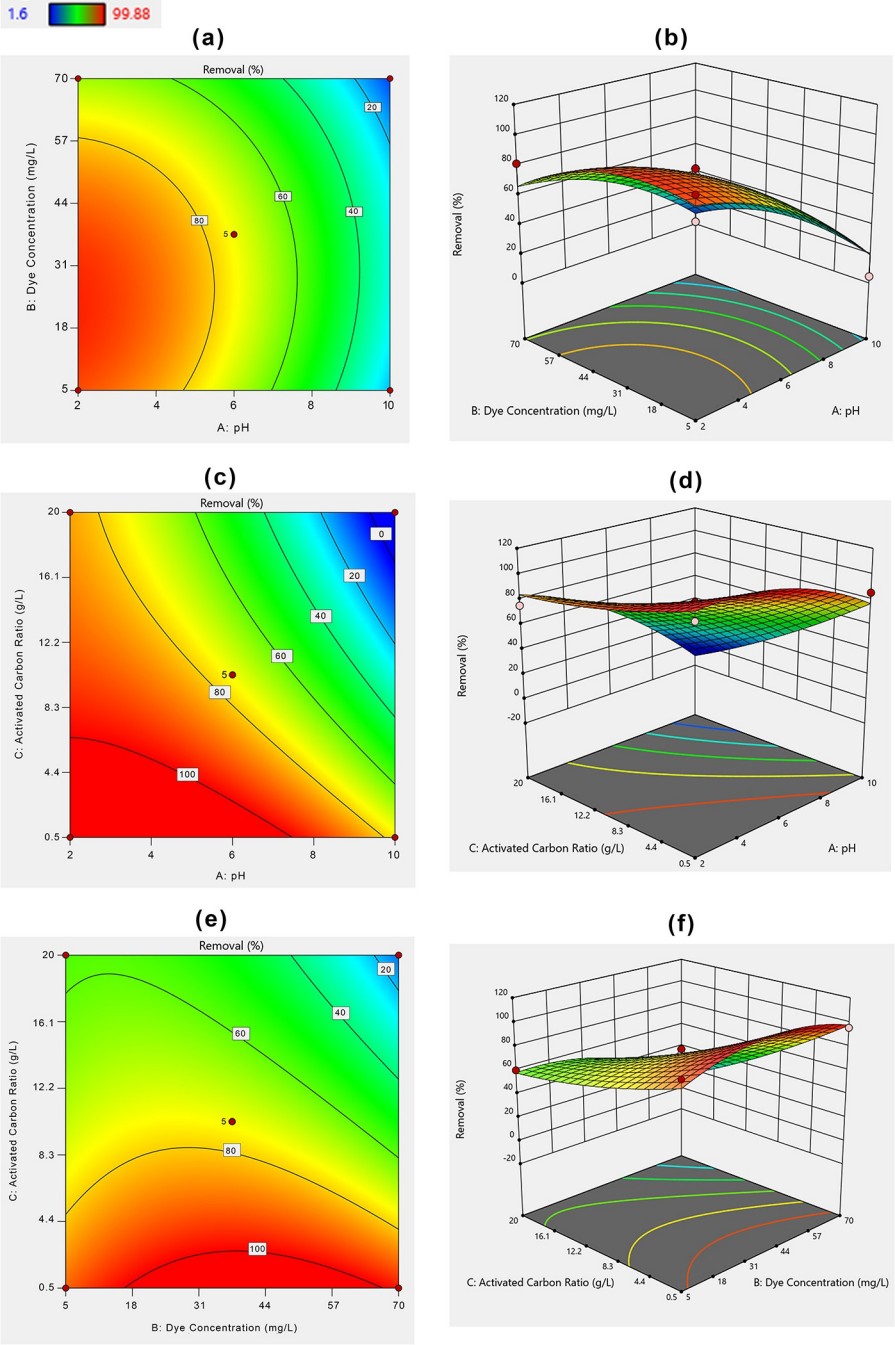
BBD 2D and 3D response plots for AR 27 adsorption using PETWBC: (a, b) solution pH; (c, d) adsorbent dose, and (e, f) initial dye concentration].

**Fig 5:**
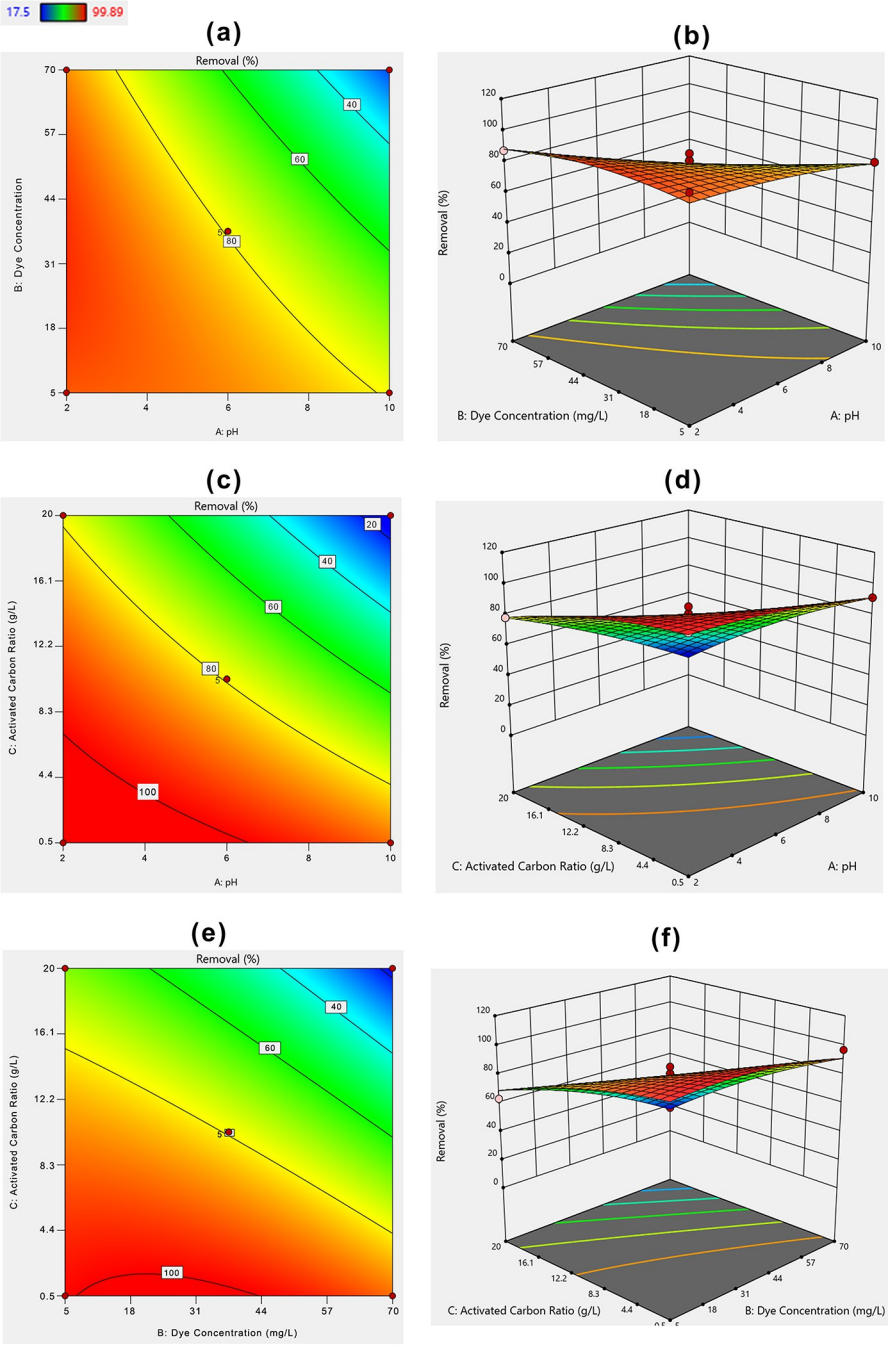
BBD 2D and 3D response plots for AR 27 adsorption using RSBC: (a, b) solution pH; (c, d) adsorbent dose, and (e, f) initial dye concentration].

For process optimization, the three factors including pH, initial MHs concentration and the adsorbent dose were applied and the studied responses are presented in S2 Table in S1 File and Figs 4 and 5, respectively. The elimination of AR 27 ranges from 4.48–99.88, and 17.5–99.89% for PETWBC and RSBC, respectively (S2 Table in S1 File). The Box–Behnken design produce 3D surface plots to understand the relation between the tested variables. This design also helps to identify the ideal experimental settings [61]. Figs 4 and 5, show the effect of solution pH, initial AR 27 concentration, and adsorbent dose on AR27 elimination efficiency using prepared PETWBC, and RSBC, respectively. Due to decreasing solution pH, the interaction between positive charge adsorbate and negatively charged adsorbent increased, consequently, the maximum removal was achieved at pH (2). A high graphene dose provides greater surface areas and huge exchangeable sites, resulting in greater adsorption performance achieve at maximum adsorbent dose. The AR 27 removal efficiency decreased with increasing dye concentrations from 5 to 70 mg/L and the highest removal was found at 5 mg /L.

The statistical association between the nominated experimental factors and the response was explained by a quadratic model with corresponding coded factors and their best fitted using the following equations.


$$\mathrm{Removal}\;(\mathrm{PETWBC}\;\%)=74.35200\;‐32.76750X1\;‐9.52500X2\;‐29.28750X3\;+3.68500X1*X2\;‐14.84000X1*X3\;‐13.21500X2*X3\;‐13.39100X1^{2}\;‐14.32600X2^{2}\;+4.77400X3^{2}$$ (11)

$$\mathrm{Removal}(\mathrm{RSBC}\;\%)=79.42000\;‐19.71250X1\;‐15.04125X2\;‐26.10125X3\;‐12.79000X1*X2\;‐13.01000X1*X3\;‐10.91250X2*X3\;‐2.99125X1^{2}‐5.52875X2^{2}‐4.57375X3^{2}$$ (12)

Analysis of variance (ANOVA) is vital for assessing the significance level of the second-order model and the result of ANOVA for AR 27 adsorption, presented in S3 and S4 Tables in S1 File, respectively, where pH and adsorbent dose show a significant effect for AR 27 removal from aqueous solution. The second-order model was statistically significant for PETWBC (P < 0.0019), and Pb (P < 0.0001) as shown in S3 and S4 Tables in S1 File, respectively. Lack of fit is more significant than the pure error in terms of the p-value (p< 0.0001). Besides, A, C, AC, BC, A^2^, and B^2^, respectively are significant model (p<0.05) terms for AR 27 adsorption using both adsorbents. Adsorbent dose and pH significantly influence AR 27 adsorption. Moreover, the *R*^*2*^ value of PETWBC = 0.94, and RSBC = 0.97 showed that 94, and 97% of the whole variability of the result was explained by this model. A good agreement was attained between experimental (R^2^) and projected (R^2^adj) results for AR 27 dye adsorption (S2 Fig in S1 File), showing that the model is a good fit, where most of the data points near the straight line. The Model F-value of PETWBC and RSBC was 11.67, and 27.84, respectively suggesting that the models are significant. There is only a 0.19, and 0.01% chance for PETWBC, and RSBC, respectively to create noise due to higher an F-value. The signal-to-noise ratio for PETWBC (11.85), and RSBC (16.94) was larger than 4 representing a satisfactory signal. Venkataraghavan et al. [62] and Oyekanmi et al. [63] use this model for dye adsorption from wastewater using bio adsorbent, respectively.

*Artificial neural network modeling*. ANNs are widely used for recording the non-linear relation between independent and dependent variables and are suitable to apply to any condition [39]. This study applies a multi-level feed-forward neural network, which is directed in the following order: input-hidden-output. Applied ANN having 60% training, 20% validation, and 20% testing networks. The input parameters (pH, adsorbent dose, and initial dye concentration) were selected for ANN, while the percentage of dye removal was selected as the output layer. The trial-and-error techniques were applied to achieve the model accuracy and validation and testing are carried out using MATLAB (R2020a).

**Fig 6:**
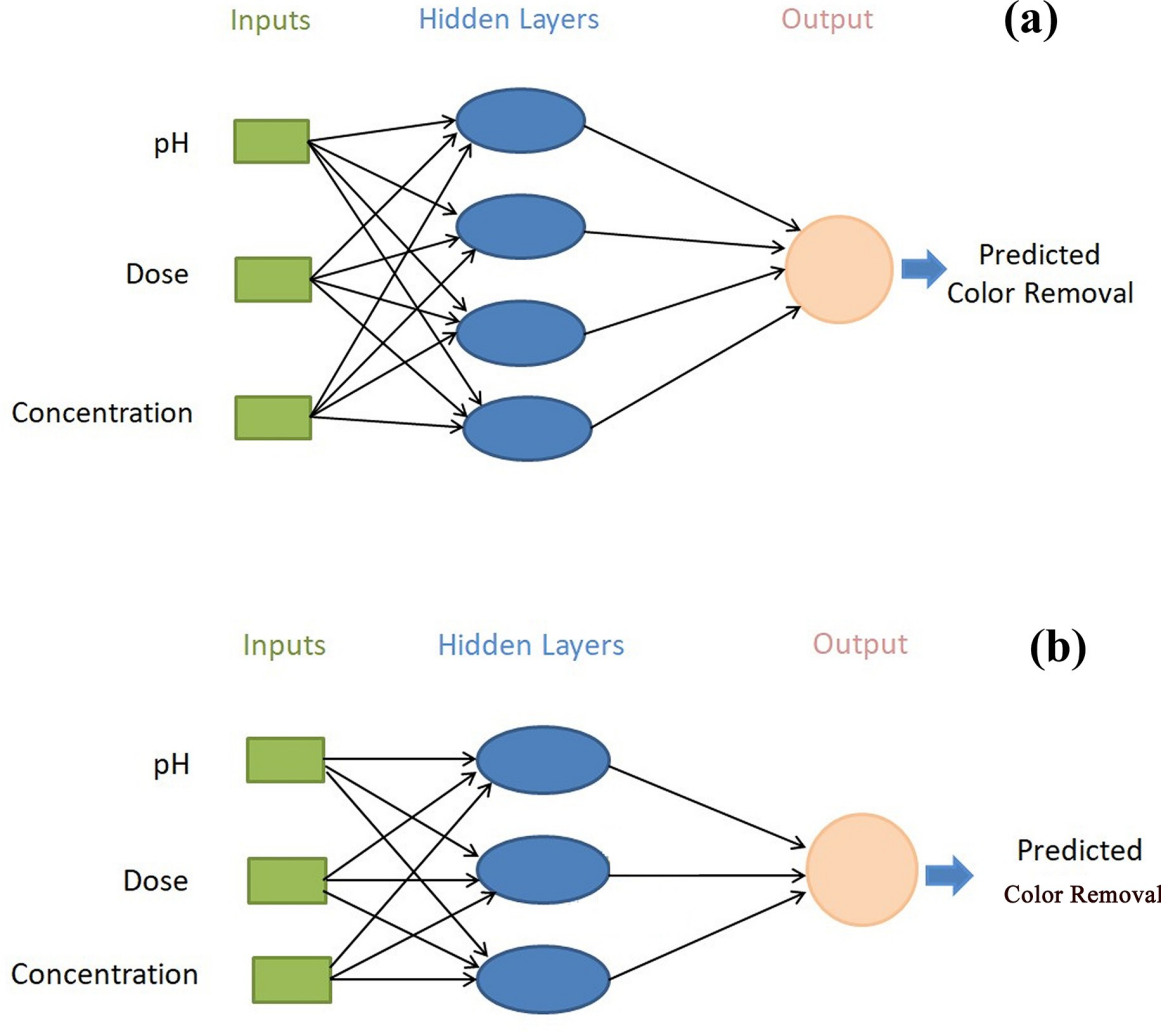
ANN network with topology for AR27 adsorption using adsorbent (a) PETWBC, and (b) RSBC.

**Fig 7:**
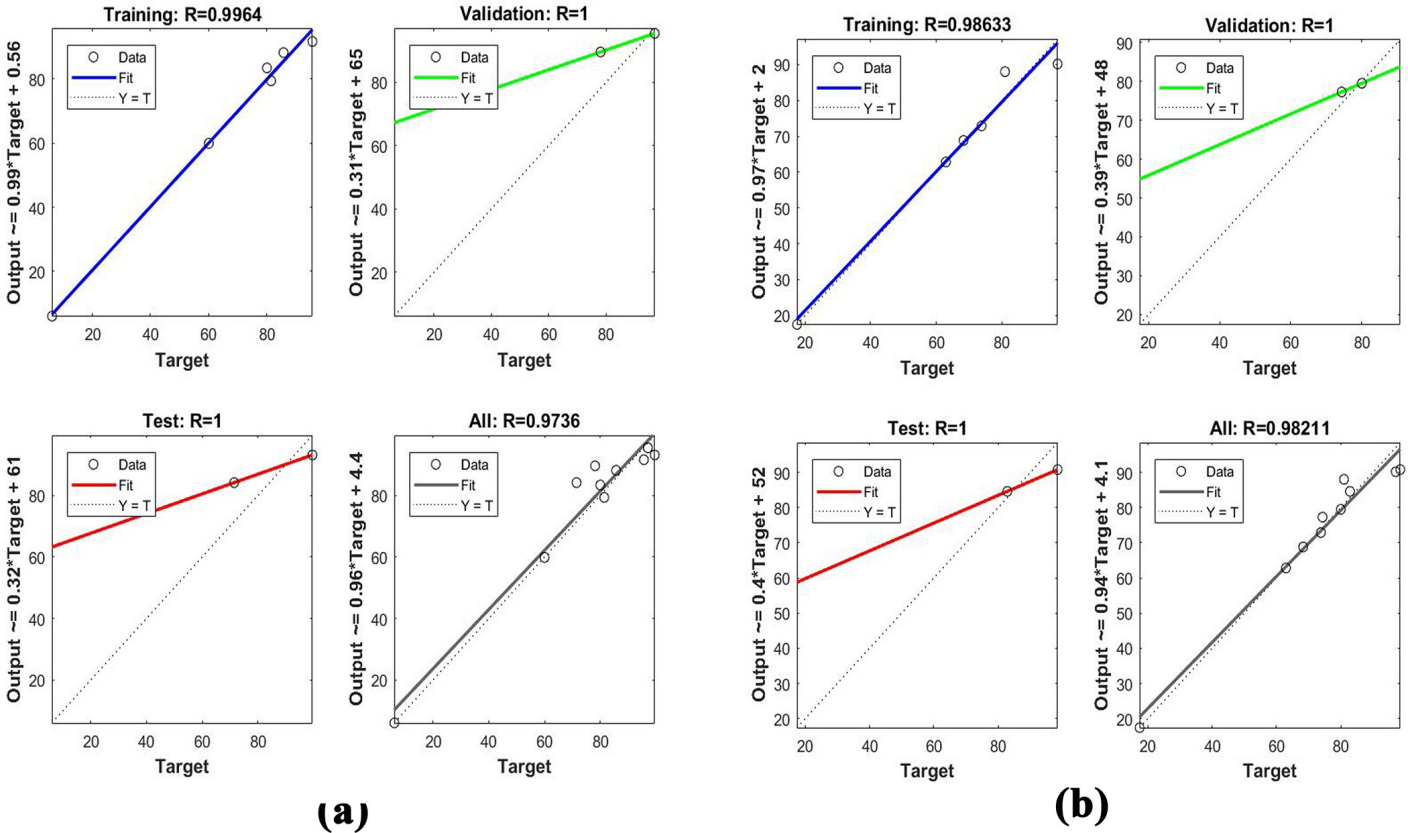
Linear fit for experimental and predicted concentration for AR 27 adsorption using ANN for (a) PETWBC, and (b) RSBC.

Fig 6 represents the topology for dye adsorption including 3:4:1, and 3:3:1 for PETWBC, and RSBC, respectively. The high and low frequencies of hidden neurons directly affect the ANN presentation and the appraisal of accuracy. So, the ideal quantities of hidden neuron selection assist to escape over and under estimation [39]. ANN performance is improved with rising neuron numbers, but the coefficients of the determinant (R^2^) did not represent the same outcome in the training phase. In the case of AR27 adsorption, all training, validation, and testing phase of tan-sigmoidal and topology was selected according to high R-value and its associated lower MSE value (S5 Table in S1 File). Good associations between experimental and ANN-predicated results (Fig 7) indicate that the ANN model was suitable for describing AR 27 adsorption using PETWBC, and RSBC. Fetimi, et al. [64] and Ahmad et al. [65] stated the same relevant result for dye adsorption modelling using ANN.

#### Real wastewater experiment

**Fig 8:**
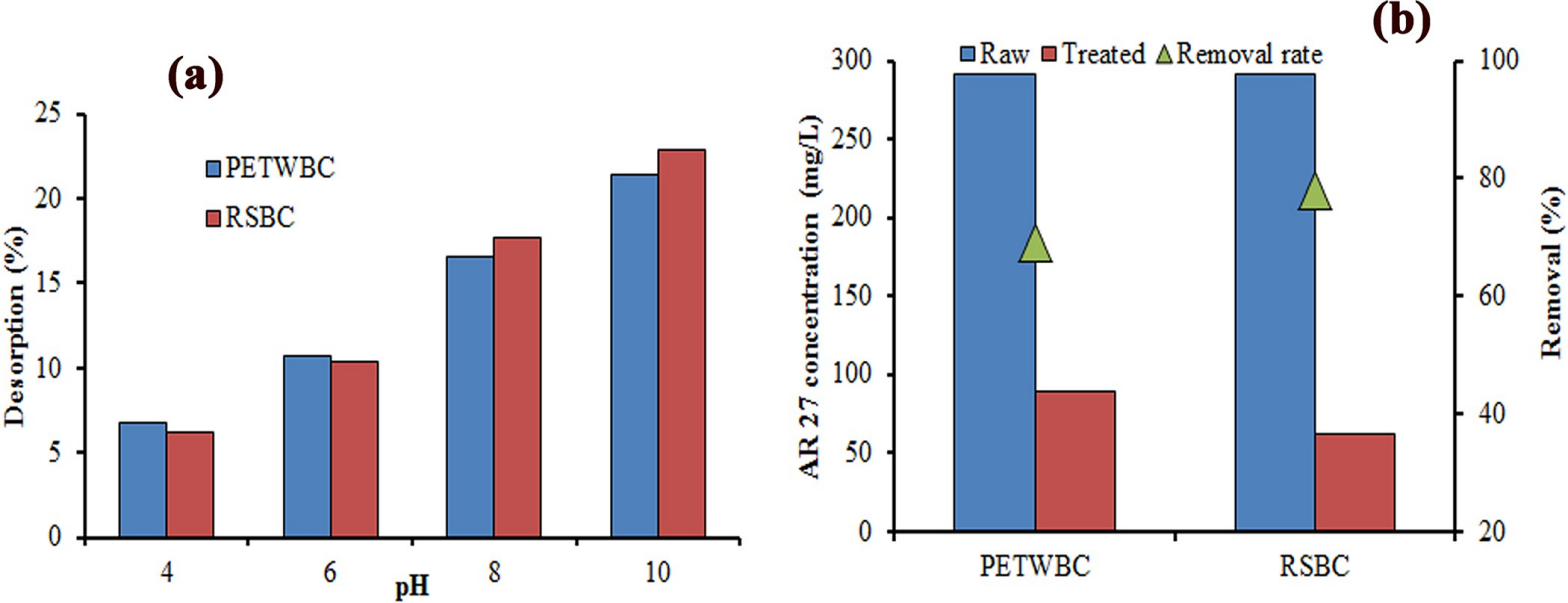
(a) Desorption experiment, and (b) Real wastewater experiment for AR 27 adsorption.

This study utilizes PETWBC and RSBC for exploring the performance of removing AR 27 from real wastewater (RWW) experiments. The physicochemical characteristics of RWW were pH (8.3), TDS (482 mg/L), EC (988 μS/cm), and salinity (0.4 ppt). The initial concentration of AR 27 in RWW was 291.5 mg/L and after the treatment at pH 2, the concentration was found 89.67, and 62.17 mg/L, for PETWBC, and RSBC respectively at 520 nm absorbance. The removal rate of AR 27 was 69, and 78%, for PETWBC, and RSBC, respectively (Fig 8B). This displays the probable uses of both adsorbents for eliminating AR 27 from industrial wastewater. Chakraborty et al. [58] found similar results for adsorption acid and basic dye using carbonized PET plastic microplastic particles.

#### Desorption study

This process is applied to assess the possibility of further contamination when the treated adsorbent comes into the environment. The desorption rate of adsorbent significantly influences by the nature of bonding (ionic bonds, Van der Waals forces or covalent) between adsorbate and adsorbent [1]. In this study, a desorption study was conducted with diverse pH values (pH 4–10). S4b Fig in S1 File shows the desorption percentage of AR 27 was very low (PETWBC = 6–22%, RSBC = 6–23%) instead of increasing pH might be the possibility of existing strong chemical bonding between AR 27 dye molecules and adsorbent (Fig 8A), confirming the eco-friendly properties. Mouni et al. [66] and Chakraborty et al. [6] provide a similar explanation in their study.

## Conclusions

This present study explores the usability of PETWBC and RSBC as potential biosorbents for AR 27 removal from simulated wastewater under batch adsorption experiments. Study finding shows that synthesize adsorbent has high performance in removing AR 27 dye from wastewater. The elimination efficiency of AR 27 decreases with rising dye concentration (PETWBC = 98–60%; and RSBC = 99–68%) and pH (PETWBC = 95–71%; and RSBC = 97–62%) while enhancing with rising temperature (PETWBC = 95–99%, and RSBC = 97–99%) and adsorbent dose (PETWBC = 6–99%, and RSBC = 17–99%). The equilibrium contact time of AR 27 was 150 min, and the optimum pH was (2). BBD and ANN models are suitable for AR 27 adsorption modelling, where both models showed a good agreement between predicted and experimental results. Pseudo-second-order was the best-matching kinetic model for AR 27adsorption data. The equilibrium data was well explained by the Freundlich isotherm model. The monolayer maximum adsorption capacity of AR 27 was 4.108, and 4.840 mg/g for PETWBC and RSBC, respectively. Thermodynamic study shows the adsorption is endothermic, physical, and spontaneous for AR 27 removal. Cost-effectiveness, obtainability, and favourable study results make PETWBC and RSBC suitable and effective adsorbents for removing dyes and other environmental pollutants. Consequently, PETWBC and RSBC could be applied for effluent treatment, where a centralized wastewater treatment system is not accessible.

## Supporting information

S1 File Supporting information for the manuscript. (DOCX)
