# Supplementary material for: Black carbon derived PET plastic bottle waste and rice straw for sorption of Acid Red 27 dye: Machine learning approaches, kinetics, isotherm and thermodynamic studies
Source: PLoS One. 2023 Aug 23;18(8):e0290471. doi: 10.1371/journal.pone.0290471 (PMC10446224; doi:10.1371/journal.pone.0290471)
Supplement: S1 File — (DOCX) [file pone.0290471.s001.docx]

**Black carbon derived PET plastic bottle waste and rice straw for sorption of Acid Red 27 dye: Machine learning approaches, kinetics, isotherm and thermodynamic studies**

Tapos Kumar Chakraborty^a*^, Lamia Tammim^a^, Khandakar Rashedul Islam^a^, Md. Simoon Nice^a^, Baytune Nahar Netema^a^, Md. Sozibur Rahman^a^ , Sujoy Sen^a^, Samina Zaman^a^, Gopal Chandra Ghosh^a^, Asadullah Munna^a^, Ahsan Habib^a^, Khadiza Tul-Coubra^a^, Himel Bosu^a^, Monishanker Halder^b^ and Md. Aliur Rahman^c^

^a^Department of Environmental Science and Technology, Jashore University of Science and Technology, Jashore 7408, Bangladesh.

^b^Department of Computer Science and Engineering, Jashore University of Science and Technology, Jashore 7408, Bangladesh.

^c^Department of Petroleum and Mining Engineering, Jashore University of Science and Technology, Jashore 7408, Bangladesh.

*Corresponding Author: E-mail: [taposchakraborty@just.edu.bd](mailto:taposchakraborty@just.edu.bd)

**Table S1** Adsorption modeling and error analysis procedure

| **Adsorption Model** | **Equation** | **Parameter** | **References** |
| --- | --- | --- | --- |
| Langmuir isotherm | $q_{e}=\frac{q_{\max} b C_{e}}{1+bC_{e}} (4)$ | Ce (mg/L)= equilibrium concentration of dye.  qe (mg/g)= amount of dye adsorbed at equilibrium.  q_max_(mg/g)=maximum monolayer coverage  b (L/mg)=Langmuir constant. | [1] |
|  | $R_{L}=\frac{1}{1+bC_{0}} (5)$ | R_L_= separation factor  unfavourable, linear, favourable, and irreversible R_L_> 1;  R_L_ = 1; 0 < R_L_<1; and R_L_ = 0, respectively. |  |
| Freundlich isotherm | $q_{e}=K_{F}C_{e}^{1/n} (6)$ | K_F_ = Freundlich constant.  n=adsorption intensity. | [2] |
| Temkin isotherm | $q_{e}=B_{T}{ln}_{A_{T}}+ B_{T}{ln}_{C_{e}}$ (7) | B_T_ (KJ/mol) = constant of adsorption heat  A_T_ (L/mg) = Temkin equilibrium constant | [3, 4] |
| Dubinin-Radushkevich isotherm | ${ln}_{q_{e}}={ln}_{q_{m} -}\beta\varepsilon^{2}$ (8)  $\varepsilon=RT\ln( 1+\frac{1}{C_{e}}$) (9)  $E=\frac{1}{\sqrt{2\beta}}$ (10) | qm= saturation capacity of theoretical isotherms (mg/g)  β (mol^2^ K/J^2^)= constant of Dubinin-Radushkevich isotherm  Ɛ = Polanyi potential (J/mol)  E (KJ/mol) =energy | [5] |
| Pseudo-first-order kinetic | $q_{t}=q_{e} \left( 1-e^{-k_{1}t} \right) (11)$ | K_1_ = pseudo-first-order rate constant (min^-1^), q_e_, and q_t_ are the quantity of dye adsorbed (mg/g) at equilibrium and time, t. | [6] |
| Pseudo-second-order kinetic | $q_{t}=\frac{K_{2}q_{e}^{2}t}{1+K_{2}q_{e}t} (12)$ | K_2_ = pseudo-second-order rate constant (g/mg/min). | [7] |
| Intraparticle diffusion | $q_{t}={K_{diff.}t}^{0.5}+C (13)$ | K_diff_. = intraparticle diffusion rate constant (mg/g min^0.5^),  C =intercept. | [8] |

**Table S2** Box–Behnken design matrix for AR 27 dye removal

|  | Factor 1 | Factor 2 | Factor 3 | Response 1 | Response 2 | Response 3 | Response 4 | Response 4 |
| --- | --- | --- | --- | --- | --- | --- | --- | --- |
| Run | A:X1 | B:X2 | C:X3 | pH | Dye Concentration | Activated Carbon Ratio | Removal | Removal |
|  |  |  |  |  | mg/L | g/L | % (PETWBC) | %  (RSBC) |
| 1 | -1 | 1 | 0 | 2 | 70 | 10.25 | 81.42 | 87.38 |
| 2 | 0 | 1 | -1 | 6 | 70 | 0.5 | 95.62 | 97.04 |
| 3 | 0 | 0 | 0 | 6 | 37.5 | 10.25 | 63.02 | 68.56 |
| 4 | 1 | 0 | -1 | 10 | 37.5 | 0.5 | 85.68 | 91.36 |
| 5 | 1 | 0 | 1 | 10 | 37.5 | 20 | 1.91 | 17.8 |
| 6 | -1 | -1 | 0 | 2 | 5 | 10.25 | 99.04 | 98.72 |
| 7 | 0 | 0 | 0 | 6 | 37.5 | 10.25 | 74.32 | 85.61 |
| 8 | 1 | -1 | 0 | 10 | 5 | 10.25 | 4.48 | 80 |
| 9 | 1 | 1 | 0 | 10 | 70 | 10.25 | 1.6 | 17.5 |
| 10 | 0 | -1 | 1 | 6 | 5 | 20 | 60.41 | 63.42 |
| 11 | 0 | 1 | 1 | 6 | 70 | 20 | 6.13 | 18.35 |
| 12 | 0 | 0 | 0 | 6 | 37.5 | 10.25 | 78.14 | 80.98 |
| 13 | 0 | 0 | 0 | 6 | 37.5 | 10.25 | 78.14 | 80.98 |
| 14 | 0 | -1 | -1 | 6 | 5 | 0.5 | 97.04 | 98.46 |
| 15 | 0 | 0 | 0 | 6 | 37.5 | 10.25 | 78.14 | 80.97 |
| 16 | -1 | 0 | -1 | 2 | 37.5 | 0.5 | 99.88 | 99.89 |
| 17 | -1 | 0 | 1 | 2 | 37.5 | 20 | 75.47 | 78.37 |

**Table S3**. ANOVA for Quadratic model for AR 27 dye removal using PETWBC

| Source | Sum of Squares | | df | | Mean Square | F-value | | p-value | |  |
| --- | --- | --- | --- | --- | --- | --- | --- | --- | --- | --- |
| Model | 19564.53 | | 9 | | 2173.84 | 11.67 | | 0.0019 | | significant |
| A-X1 | 8589.67 | | 1 | | 8589.67 | 46.09 | | 0.0003 | |  |
| B-X2 | 725.81 | | 1 | | 725.81 | 3.89 | | 0.0890 | |  |
| C-X3 | 6862.06 | | 1 | | 6862.06 | 36.82 | | 0.0005 | |  |
| AB | 54.32 | | 1 | | 54.32 | 0.2915 | | 0.6060 | |  |
| AC | 880.90 | | 1 | | 880.90 | 4.73 | | 0.0662 | |  |
| BC | 698.54 | | 1 | | 698.54 | 3.75 | | 0.0941 | |  |
| A² | 755.03 | | 1 | | 755.03 | 4.05 | | 0.0840 | |  |
| B² | 864.14 | | 1 | | 864.14 | 4.64 | | 0.0683 | |  |
| C² | 95.96 | | 1 | | 95.96 | 0.5150 | | 0.4962 | |  |
| Residual | 1304.45 | | 7 | | 186.35 |  | |  | |  |
| Lack of Fit | 1132.99 | | 3 | | 377.66 | 8.81 | | 0.0309 | | significant |
| Pure Error | 171.46 | | 4 | | 42.87 |  | |  | |  |
| Cor Total | 20868.98 | | 16 | |  |  | |  | |  |
| Std. Dev. | | 13.65 | |  | | | R² | | 0.9375 | |
| Mean | | 63.56 | |  | | | Adjusted R² | | 0.8571 | |
| C.V. % | | 21.48 | |  | | | Predicted R² | | 0.1185 | |
|  | |  | |  | | | Adeq Precision | | 11.8540 | |

**Table S4**. ANOVA for Quadratic model for AR 27 dye removal using RSBC

| Source | Sum of Squares | | df | | Mean Square | F-value | | p-value | |  |
| --- | --- | --- | --- | --- | --- | --- | --- | --- | --- | --- |
| Model | 12458.56 | | 9 | | 1384.28 | 27.84 | | 0.0001 | | significant |
| A-X1 | 3108.66 | | 1 | | 3108.66 | 62.52 | | < 0.0001 | |  |
| B-X2 | 1809.91 | | 1 | | 1809.91 | 36.40 | | 0.0005 | |  |
| C-X3 | 5450.20 | | 1 | | 5450.20 | 109.62 | | < 0.0001 | |  |
| AB | 654.34 | | 1 | | 654.34 | 13.16 | | 0.0084 | |  |
| AC | 677.04 | | 1 | | 677.04 | 13.62 | | 0.0078 | |  |
| BC | 476.33 | | 1 | | 476.33 | 9.58 | | 0.0174 | |  |
| A² | 37.67 | | 1 | | 37.67 | 0.7577 | | 0.4129 | |  |
| B² | 128.70 | | 1 | | 128.70 | 2.59 | | 0.1517 | |  |
| C² | 88.08 | | 1 | | 88.08 | 1.77 | | 0.2249 | |  |
| Residual | 348.04 | | 7 | | 49.72 |  | |  | |  |
| Lack of Fit | 184.51 | | 3 | | 61.50 | 1.50 | | 0.3419 | | not significant |
| Pure Error | 163.53 | | 4 | | 40.88 |  | |  | |  |
| Cor Total | 12806.60 | | 16 | |  |  | |  | |  |
| Std. Dev. | | 7.05 | |  | | | R² | | 0.9728 | |
| Mean | | 73.26 | |  | | | Adjusted R² | | 0.9379 | |
| C.V. % | | 9.63 | |  | | | Predicted R² | | 0.7495 | |
|  | |  | |  | | | Adeq Precision | | 16.9428 | |

**Table S5**. Comparison of MB color removal prediction using tan-sigmoidal and Log-sigmoidal transfer functions

| Types | Topo-logy | Tan-sigmoidal | | | | | Log-sigmoidal | | | | |
| --- | --- | --- | --- | --- | --- | --- | --- | --- | --- | --- | --- |
|  |  | MSE | R | | | | MSE | R | | | |
|  |  |  | Tr | Val | Test | All |  | Tr | Val | Test | All |
| PETWBC | 3:2:1 | 7.81 | 0.930 | 1 | 1 | 0.935 | 20.14 | 0.96 | 1 | 1 | 0.99 |
|  | 3:3:1 | 33.59 | 0.97 | 1 | 1 | 0.9 | 24.99 | 0.99 | 1 | 1 | 0.99 |
|  | 3:4:1 | 0.03 | 0.97 | 1 | 1 | 0.97 | 67.28 | 0.99 | 1 | 1 | 0.97 |
|  | 3:5:1 | 37.52 | 0.97 | 1 | 1 | 0.99 | 99.91 | 0.88 | 1 | 1 | 0.87 |
|  | 3:6:1 | 440.02 | 1 | 1 | 1 | 0.87 | 117.09 | 0.99 | 1 | 1 | 0.96 |
|  | 3:7:1 | 15.50 | 0.87 | 1 | 1 | 0.88 | 103.05 | 0.97 | 1 | 1 | 0.94 |
| RBSC | 3:2:1 | 5.58 | 0.98 | 1 | 1 | 0.87 | 2.31 | 0.68 | 1 | 1 | 0.96 |
|  | 3:3:1 | 4.47 | 0.99 | 1 | 1 | 0.98 | 44.84 | 0.47 | 1 | 1 | 0.56 |
|  | 3:4:1 | 39.11 | 0.97 | 1 | 1 | 0.96 | 12.54 | 0.60 | 1 | 1 | 0.60 |
|  | 3:5:1 | 0.87 | 0.87 | 1 | 1 | 0.97 | 89.02 | 0.94 | 1 | 1 | 0.93 |
|  | 3:6:1 | 99.5 | 0.53 | 1 | 1 | 0.51 | 806.59 | 0.93 | 1 | 1 | 0.86 |
|  | 3:7:1 | 860.58 | 0.99 | 1 | 1 | 0.83 | 516.88 | 0.98 | 1 | 1 | 0.94 |

**
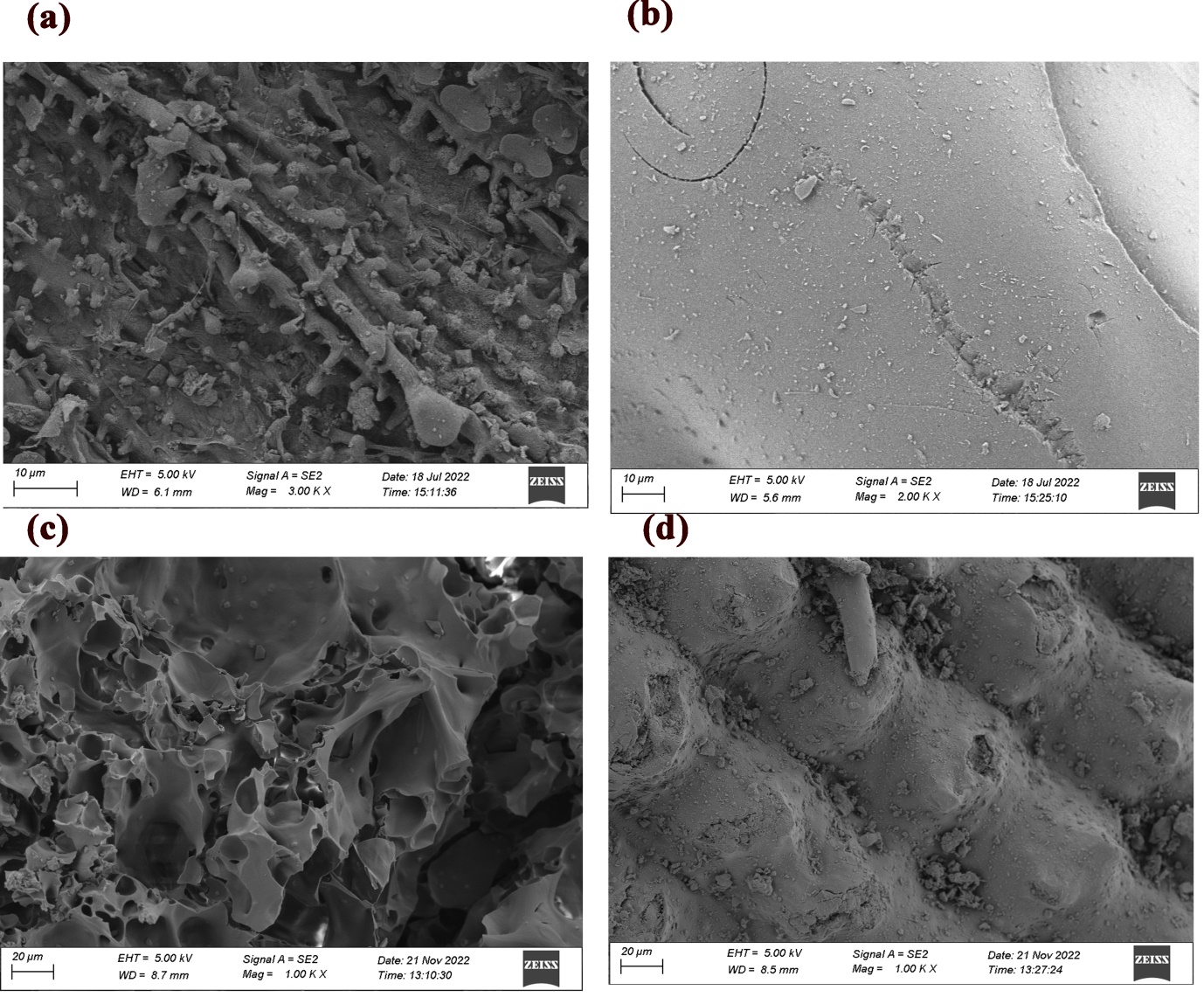
**

Fig S1. PETWBC (a) before adsorption, (b) after adsorption; RSBC (c) before adsorption, (d) after adsorption.


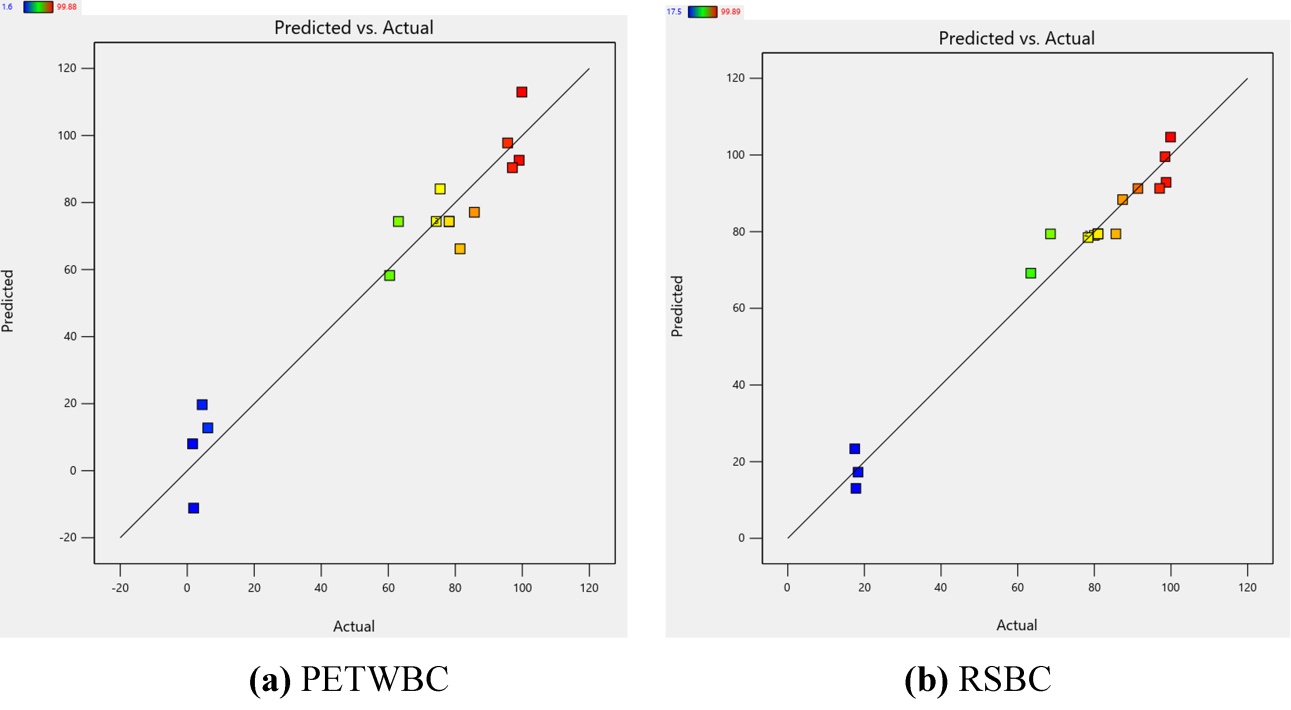


Fig S2. Predicted Vs actual for AR 27 dye adsorption derived from BBD model.

**References**

1. Langmuir, I. The constitution and fundamental properties of solids and liquids. II. Liquids. J. Am. Chem. Soc. 1917; 39 (9), 1848-1906, https://doi.org/10.1021/ja02254a006.
2. Freundlich, H.M.F. Over the adsorption in solution. J. Phys. Chem. 1906; 57 (385471), 1100-1107.
3. Mostafapour, F. K., Mahvi, A. H., Khatibi, A. D., Saloot, M. K., Mohammadzadeh, N., and Balarak, D. Adsorption of lead (II) using bioadsorbent prepared from immobilized Gracilaria corticata algae: thermodynamics, kinetics and isotherm analysis. Desalin. Water Treat. 2022; 265, 103-113. https://doi.org/10.5004/dwt.2022.28627.
4. Khatibi, A. D., Yilmaz, M., Mahvi, A. H., Balarak, D., and Salehi, S. Evaluation of surfactant-modified bentonite for Acid Red 88 dye adsorption in batch mode: kinetic, equilibrium, and thermodynamic studies. Desalin. Water Treat. 2022; 271, 48-57. https://doi.org/10.5004/dwt.2022.28812.
5. Mostafapour, F. K., Yilmaz, M., Mahvi, A. H., Younesi, A., Ganji, F., and Balarak, D. Adsorptive removal of tetracycline from aqueous solution by surfactant-modified zeolite: equilibrium, kinetics and thermodynamics. Desalin. Water Treat. 2022; 247, 216-228. https://doi.org/10.5004/dwt.2022.27943.
6. Lagergren, S. Zur theorie der sogenannten adsorption geloster stoffe. Kungl. Svenska. Vetenskapsakad. Handl. 1898; 24, 1-39.
7. Ho, Y.S., Mckay, G. The kinetics of sorption of divalent metal ions onto sphagnum moss peat. Water Res. 2000; 34, 735-742, https://doi.org/10.1016/S0043-1354(99)00232-8.
8. Weber, G.R., Morris, J.C. Kinetics of adsorption on carbon from solutions. J. Sanit. Eng. Div. 1963; 89 (2), 31-59, https://doi.org/10.1061/JSEDAI.0000430.
